# Rapidly Progressing Radicular Cyst in an Endodontically Treated Tooth: Diagnostic Challenges, Advanced Imaging, and Surgical Management

**DOI:** 10.1002/ccr3.70409

**Published:** 2025-04-09

**Authors:** Saeed Asgary, Nafise Shamloo

**Affiliations:** ^1^ Iranian Centre for Endodontic Research, Research Institute of Dental Sciences Shahid Beheshti University of Medical Sciences Tehran Iran; ^2^ Department of Oral and Maxillofacial Pathology, School of Dentistry Shahid Beheshti University of Medical Sciences Tehran Iran

**Keywords:** calcium derivative, calcium‐enriched mixture, endodontics, mineral trioxide aggregate, radicular cyst, tooth pulp disease, tooth resorption, volume CT

## Abstract

Radicular cysts in teeth that have undergone root canal therapy (RCT) are uncommon but clinically important, often presenting unique challenges for diagnosis. Although rare, radicular cysts can progress rapidly, leading to symptoms, such as discomfort or swelling shortly after the initial treatment. In this case, a 29‐year‐old female experienced mild discomfort localized to the maxillary right first premolar, which had received RCT several months prior. The patient had no history of systemic conditions or chronic infections. Upon clinical examination, tenderness was noted in the periapical region; however, conventional radiographs did not reveal detectable lesions. CBCT detected a well‐defined cystic lesion at the distal aspect of the buccal root apex. Surgical intervention was performed, which included cystectomy and root‐end filling with calcium‐enriched mixture cement. Histopathological analysis confirmed the diagnosis of a benign radicular cyst with a fibrous capsule and squamous lining, indicating no malignancy. The postoperative recovery was smooth, and a follow‐up 1 year later showed a normal appearance of the periodontal ligament in radiographs, confirming the success of the treatment and retention of the tooth. This case highlights the diagnostic benefits of CBCT in detecting periapical lesions that may not be visible on conventional radiographs. It emphasizes the effectiveness of surgical management in resolving such endodontic lesions while maintaining tooth function.

## Graphical abstract

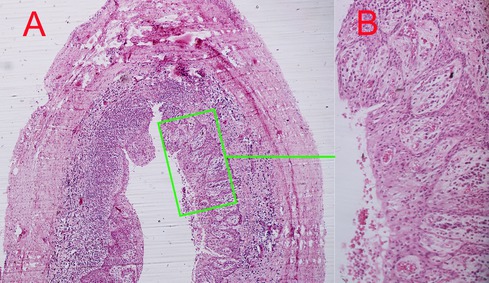

**Summary**

- Radicular cysts in endodontically treated teeth, though rare, necessitate accurate diagnosis using advanced imaging, such as CBCT.
- Histopathological examination confirms their nature, distinguishing them from other periapical conditions.
- Surgical intervention, including cystectomy and root‐end filling, ensures successful resolution and long‐term tooth functionality, highlighting the importance of multidisciplinary approaches for optimal outcomes.

## 1 Introduction

Periapical cysts, comprising 52%–68% of cystic jaw lesions, are the most common odontogenic cysts [1]. These inflammatory lesions are typically located at the apex of non‐vital teeth and are characterized by an epithelium‐lined cavity. The epithelium often originates from the rests of Malassez but may also derive from crevicular epithelium or sinus lining [1]. Periapical cysts are classified into two variations: true cysts, which are entirely separated from the tooth apex, and pocket/bay cysts, which are connected to the root canal system [2]. True cysts require surgical removal, whereas pocket cysts often resolve with conventional endodontic therapy. Histologically, these cysts exhibit fibrous connective tissue lined by stratified squamous epithelium, with features, such as cholesterol clefts and desquamated epithelial cells [2]. Although asymptomatic in most cases, large cysts can cause swelling, pain, or tooth mobility, underscoring the need for timely diagnosis and intervention [3].

Persistent periapical radiolucency following endodontic procedures is a diagnostic challenge. Although many lesions resolve with adequate therapy, unresolved or enlarging radiolucencies may indicate the presence of cysts, granulomas, or other periapical pathologies [4]. True periapical cysts, in particular, resist conventional therapy and necessitate surgical management. These lesions are typically linked to chronic inflammation from pulp necrosis or trauma and can progressively destroy surrounding bone if left untreated.

Conventional radiographs often fail to detect small or overlapping periapical lesions, making diagnosis difficult. Cone‐beam computed tomography (CBCT) provides superior diagnostic accuracy, offering three‐dimensional imaging that enables precise localization and characterization of lesions [5, 6]. CBCT is particularly useful for assessing the extent of bone destruction, cortical plate involvement, and lesion margins, which are crucial for treatment planning in cases of persistent radiolucency.

This case report aimed to describe the diagnostic and management challenges of a true periapical cyst associated with an endodontically treated tooth. The report highlights the role of CBCT in identifying lesions undetectable by conventional radiography and emphasizes the importance of histopathological analysis and surgical intervention using endodontic biomaterials in ensuring successful outcomes.

## 2 Case History Examination

**FIGURE 1:**
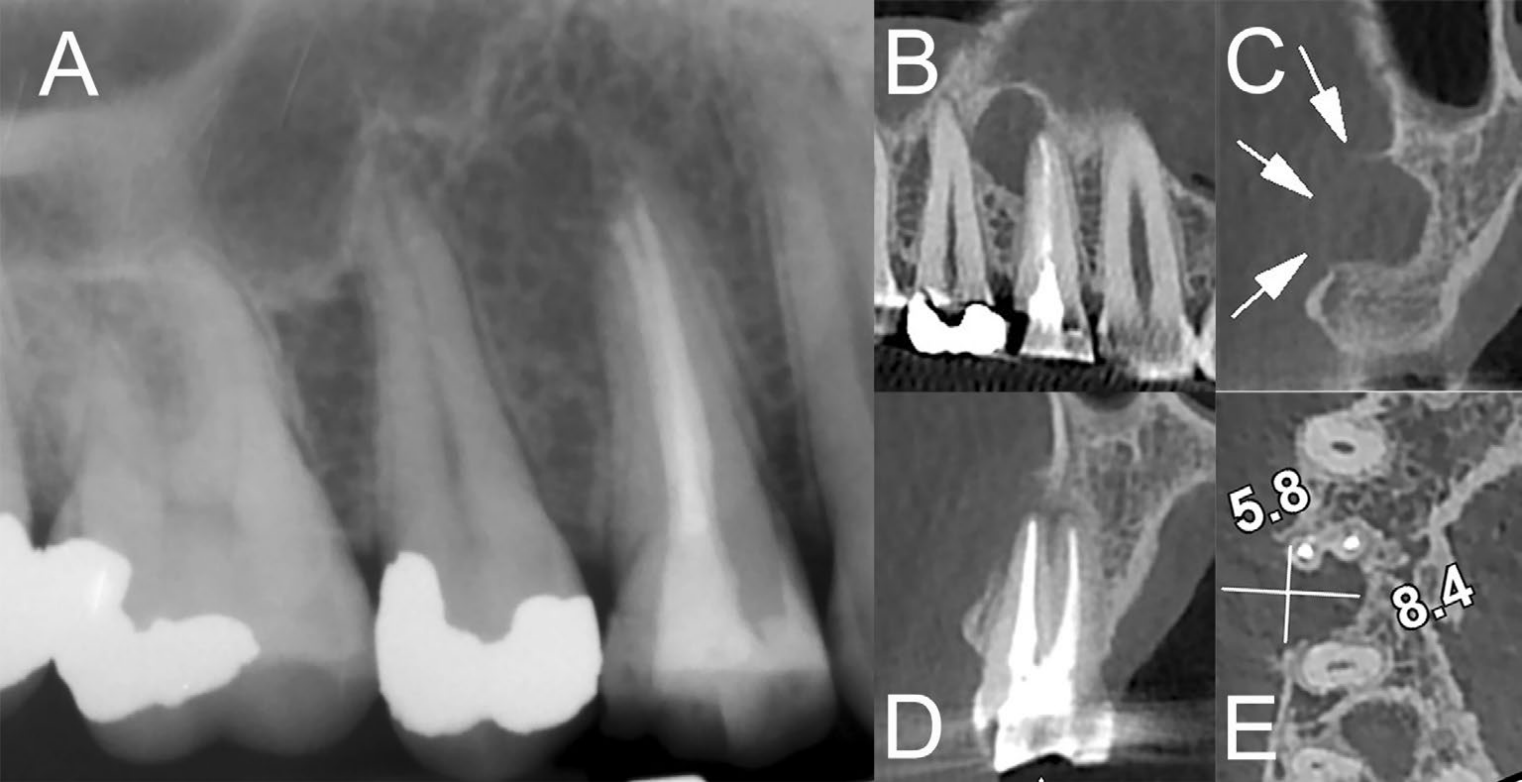
Preoperative radiographic findings. (A) Conventional periapical radiograph of the maxillary right first premolar, showing no apparent lesion. (B–E) Three‐dimensional sagittal, coronal, and axial CBCT views revealing a well‐defined, radiolucent cystic lesion at the distal aspect of the buccal root apex (~6 × 8 mm), with evidence of cortical plate thinning (white arrows).

A 29‐year‐old female presented with mild discomfort localized to the maxillary right first premolar (#14), which had undergone root canal treatment (RCT) approximately 6 months earlier. She had no history of systemic diseases or chronic infections, which are common predisposing factors for such lesions. The patient reported no history of acute pain, swelling, or systemic symptoms. Clinical examination revealed localized tenderness to palpation at the periapical region of the treated tooth. A palpable lesion was noted, but conventional periapical radiographs failed to reveal any abnormalities (Figure 1A). To further investigate, CBCT imaging was performed, which demonstrated a well‐defined, radiolucent cystic lesion measuring approximately 6 × 8 mm at the distal aspect of the buccal root apex. The lesion exhibited cortical plate thinning/bulging (Figure 1B–E).

Based on the clinical presentation and CBCT findings, a diagnosis of a persistent periapical lesion was made, suggestive of a radicular cyst. Surgical management was planned. The patient's medical history was unremarkable, with no known allergies or contraindications to treatment. Written informed consent was obtained after explaining the risks, benefits, and alternative options.

## 3 Methods

**FIGURE 2:**
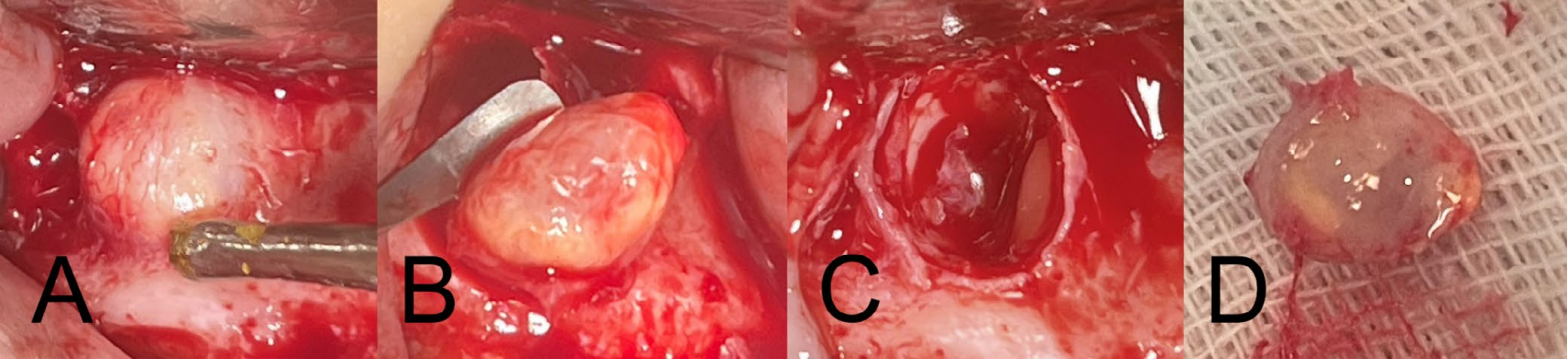
Intraoperative findings and surgical procedure. (A) Reflection of the mucoperiosteal flap revealing a bulging lesion at the buccal root apex. (B) Periapical curettage in progress, removing the lesion. (C) Post‐cystectomy view of the surgical site, demonstrating complete removal of the lesion. (D) Enucleation of the radicular cyst, illustrating the excised cystic lesion.

The procedure was performed under local anesthesia with 2% lidocaine containing epinephrine (1:80,000). Preoperative preparations included premedication with ibuprofen 400 mg and a 0.2% chlorhexidine mouth rinse. A rectangular full‐thickness mucoperiosteal flap was carefully raised, providing access to the periapical area. Upon reflection, a bulging lesion was observed at the apex of the buccal root (Figure 2A). Periapical curettage was performed to remove the lesion (Figure 2B), and the cystic lesion was completely enucleated via cystectomy (Figure 2C,D). The excised tissue was immediately fixed in 10% neutral‐buffered formalin and sent for histopathological evaluation.

Root‐end resection was performed at a ~2 mm level, and the root‐end cavity was prepared with an ultrasonic retro‐tip (Joya Electronics, Tehran, Iran). A biocompatible root‐end filling material, calcium‐enriched mixture (CEM) cement (BioniqueDent, Tehran, Iran), was used to achieve an optimal apical seal. The surgical site was closed using 4‐0 resorbable sutures to ensure adequate healing.

## 4 Results

**FIGURE 3:**
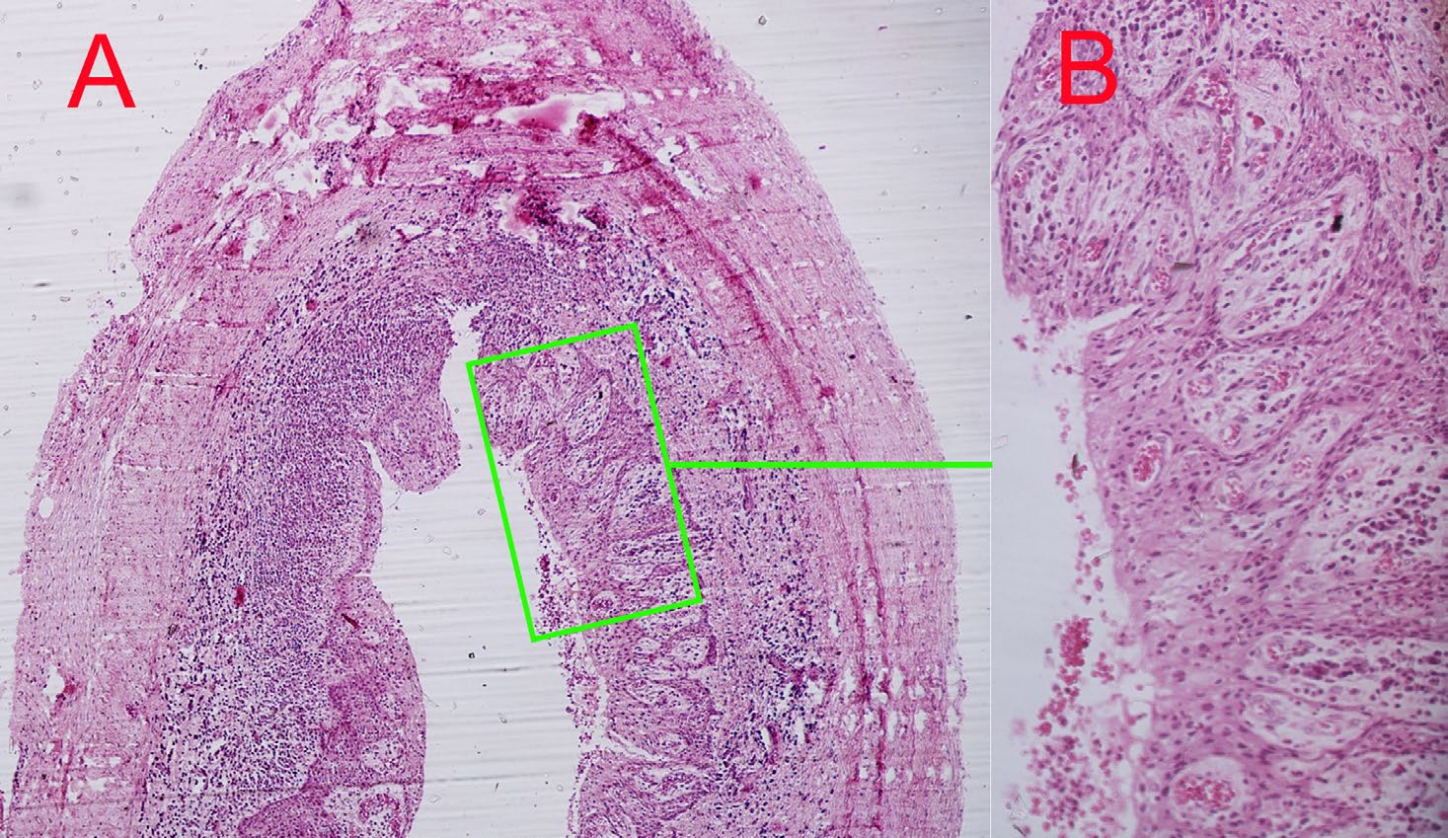
Histopathological analysis of the excised lesion. Histological section showing the fibrous connective tissue capsule lined with stratified squamous epithelium (H&E stain, original magnification ×40). (B) Higher magnification image illustrating stratified squamous epithelium (×100).

Histopathological analysis confirmed the diagnosis of a true radicular cyst. The lesion displayed a fibrous connective tissue capsule lined by stratified squamous epithelium. No evidence of malignancy, acute infection, or atypical cellular changes was observed (Figure 3A,B).

**FIGURE 4:**
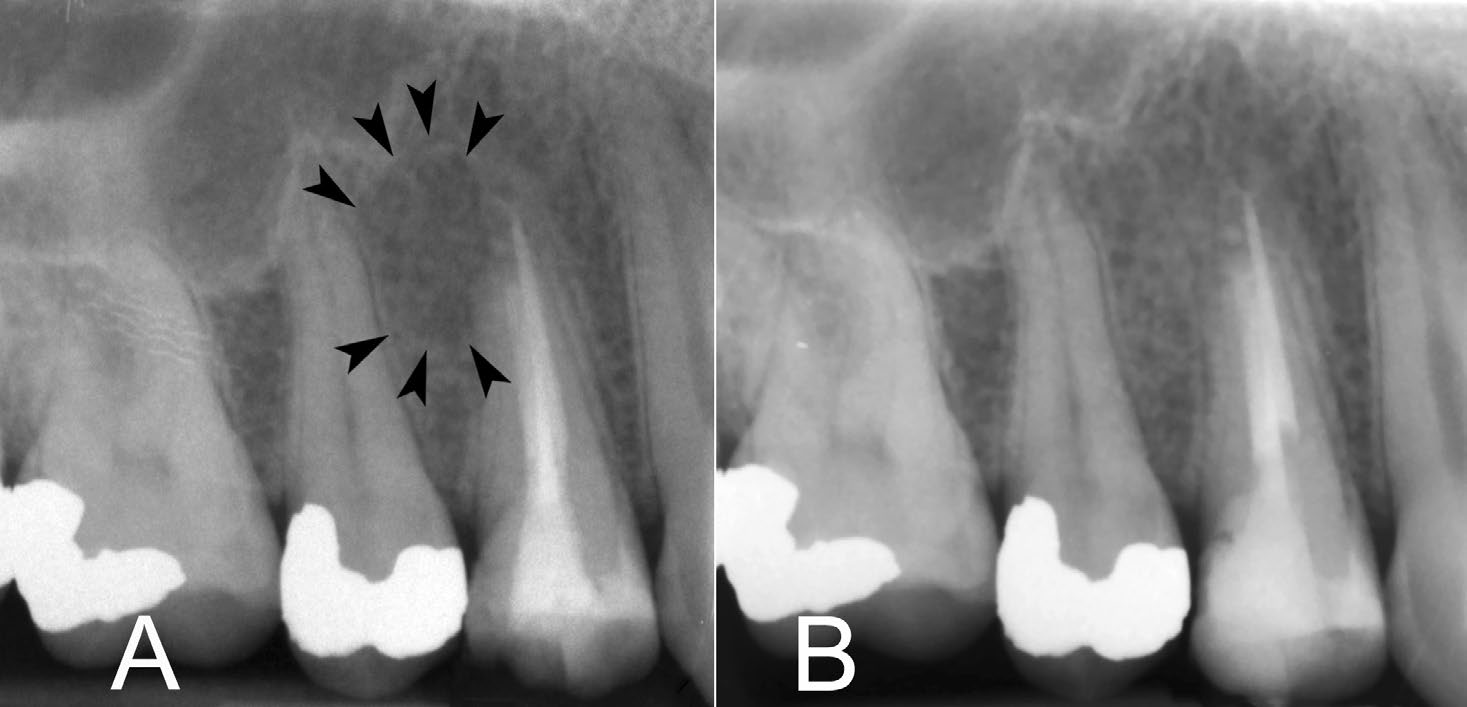
Follow‐up periapical radiographs. (A) Immediate postoperative radiograph; the lesion's borders now slightly visible (indicated by black arrowheads). (B) One‐year postoperative periapical radiograph demonstrating complete resolution of the cystic lesion and normal periodontal ligament space.

The patient experienced an uneventful recovery, with no postoperative complications (Figure 4A). At the 1‐year follow‐up, the patient reported no discomfort, and clinical examination showed complete healing of the surgical site. Radiographic evaluation demonstrated resolution of the cystic lesion, reestablishment of normal periodontal ligament space (Figure 4B), and preservation of the treated tooth in functional occlusion.

## 5 Discussion

This case underscores the complexity and clinical significance of diagnosing and managing radicular cysts in endodontically treated teeth. Radicular cysts, though the most common odontogenic cysts, rarely exhibit rapid progression or conspicuous symptoms. Their pathogenesis is linked to chronic inflammation, which stimulates the proliferation of epithelial cell rests of Malassez within the periapical region [1]. This inflammatory cascade often follows pulp necrosis or inadequate endodontic treatment, emphasizing the need for meticulous therapeutic protocols to prevent or address such complications. In this case, the rapid progression of the cyst may be attributed to persistent low‐grade inflammation following incomplete resolution of periapical pathology after initial RCT. The absence of predisposing factors, such as systemic conditions or chronic infections suggests that localized inflammatory mediators and epithelial cell proliferation played a key role in the cyst's development/progression.

Conventional radiographs are often insufficient for identifying small or overlapping periapical lesions because of their two‐dimensional limitations. In this case, initial periapical radiographs failed to clearly detect the cystic lesion despite clinical symptoms. CBCT imaging provided critical diagnostic clarity by delineating the lesion's size, location, and extent, including cortical plate thinning and buccal root apex involvement. CBCT's superior spatial resolution and three‐dimensional imaging capabilities enable the differentiation of cystic and non‐cystic lesions, which is vital for treatment planning. Moreover, CBCT facilitates the assessment of surgical anatomy, including root morphology and proximity to vital structures, reducing the risk of intraoperative complications.

Histopathological evaluation remains the gold standard for confirming the diagnosis of periapical cysts and distinguishing them from other odontogenic or non‐odontogenic lesions [2]. The findings in this case—a fibrous connective tissue capsule lined by stratified squamous epithelium—are characteristic of true radicular cysts. The absence of malignant or atypical features further validated the benign nature of the lesion.

In cases of persistent periapical lesions, such as radicular cysts, several treatment options exist, each with its own advantages and limitations. Nonsurgical endodontic retreatment is often considered the first line of management, particularly for lesions that may represent residual infection or inadequate initial RCT [7]. This approach involves reinstrumentation, disinfection, and obturation of the root canal system, which can resolve periapical pathologies. However, nonsurgical retreatment is less effective for true radicular cysts, which are entirely separated from the root canal system and require surgical intervention for complete removal [8]. Another alternative is marsupialization, a conservative surgical technique that involves creating a window in the cyst wall to reduce its size and promote healing. Although this approach is less invasive, it is typically reserved for large cysts where complete enucleation may risk damaging adjacent structures [9]. In contrast, surgical endodontics, including cystectomy and root‐end resection, offers a definitive solution for true radicular cysts, as demonstrated in this case. The success of surgical management is further enhanced by the use of advanced biomaterials, such as CEM cement or mineral trioxide aggregate (MTA), which provide excellent sealing properties and promote cementogenesis and periapical healing [10]. CEM cement was selected for its excellent sealing properties, biocompatibility, and ability to promote periapical healing through mineralized tissue formation, that is, cementogenesis [11]. Compared with other biomaterials, such as MTA, CEM cement offers similar clinical outcomes [12] with the added advantage of easier handling and shorter setting times [11]. Although nonsurgical approaches may suffice for certain lesions, surgical intervention remains the gold standard for true radicular cysts, ensuring complete removal of the pathological tissue and long‐term preservation of tooth function.

A meticulous surgical protocol was pivotal to the successful management of this case. Preoperative measures, including the use of chlorhexidine and ibuprofen, minimized infection and inflammation, whereas a carefully raised mucoperiosteal flap provided adequate access to the lesion. Complete enucleation of the cyst, combined with thorough irrigation and debridement, eliminated the pathological tissue and inflammatory mediators, creating a conducive environment for healing.

The 1‐year follow‐up demonstrated complete resolution of the cystic lesion and preservation of the treated tooth's function, affirming the success of the surgical approach. Although quantitative data on long‐term prognosis are limited in this single case, studies suggest that surgical endodontics with biocompatible materials, such as CEM cement achieve 93% success in managing persistent apical periodontitis [13]. This outcome aligns with the evidence suggesting that surgical endodontics, when appropriately executed, can achieve high success rates in managing persistent periapical lesions.

This case highlights several key lessons for clinical practice. First, it underscores the importance of early detection of persistent periapical lesions, particularly in endodontically treated teeth. Clinicians should maintain a high index of suspicion for cystic lesions in cases where symptoms persist despite normal conventional radiographs. Second, the use of advanced imaging modalities, such as CBCT should be considered in such scenarios, as it provides superior diagnostic accuracy and aids in treatment planning. Third, surgical intervention with biocompatible materials, such as CEM cement or MTA should be prioritized for true radicular cysts, as these biomaterials promote healing and reduce the risk of reinfection. Finally, this case emphasizes the need for a multidisciplinary approach, combining advanced imaging, histopathological analysis, and surgical expertise, to achieve optimal outcomes in complex periapical pathologies. By integrating these recommendations into clinical practice, dentists can improve the early diagnosis and management of persistent periapical lesions, ultimately enhancing patient outcomes and preserving tooth function.

Although the outcome in this case was favorable, the findings are limited to a single patient. Future studies with larger cohorts are needed to establish the long‐term efficacy of advanced biomaterials, such as CEM cement in managing persistent periapical lesions. Additionally, incorporating patient‐reported outcomes into clinical assessments can provide a more holistic evaluation of treatment success.

## 6 Conclusion

In conclusion, this case reinforces the critical role of CBCT and histopathological analysis in diagnosing periapical lesions and highlights the efficacy of surgical endodontics combined with advanced biomaterials for achieving favorable outcomes. The integration of these modalities ensures accurate diagnosis, effective treatment, and long‐term preservation of affected teeth.

## Author Contributions

**Saeed Asgary:** conceptualization, data curation, formal analysis, investigation, methodology, software, supervision, validation, visualization, writing – original draft, writing – review and editing. **Nafise Shamloo:** data curation, formal analysis, software, validation, visualization, writing – review and editing.

## Consent

Written informed consents were obtained from the patients to publish this case series in accordance with the journal's patient consent policy.

## Conflicts of Interest

The authors declare no conflicts of interest.

## Data Availability

The data used to support the findings of this study are included within the article.
